# Truncation of C-terminal 20 amino acids in PA-X contributes to adaptation of swine influenza virus in pigs

**DOI:** 10.1038/srep21845

**Published:** 2016-02-25

**Authors:** Guanlong Xu, Xuxiao Zhang, Yipeng Sun, Qinfang Liu, Honglei Sun, Xin Xiong, Ming Jiang, Qiming He, Yu Wang, Juan Pu, Xin Guo, Hanchun Yang, Jinhua Liu

**Affiliations:** 1 Key Laboratory of Animal Epidemiology and Zoonosis, Ministry of Agriculture, College of Veterinary Medicine, and State Key Laboratory of Agrobiotechnology, China Agricultural University, Beijing, 100193, China; 2 Department of Avian Infectious Disease, Shanghai Veterinary Research Institute, Chinese Academy of Agricultural Sciences, Shanghai, Innovation Team for Pathogen Ecology Research on Animal Influenza Virus, Shanghai, 200241, China

## Abstract

The PA-X protein is a fusion protein incorporating the N-terminal 191 amino acids of the PA protein with a short C-terminal sequence encoded by an overlapping ORF (X-ORF) in segment 3 that is accessed by + 1 ribosomal frameshifting, and this X-ORF exists in either full length or a truncated form (either 61-or 41-condons). Genetic evolution analysis indicates that all swine influenza viruses (SIVs) possessed full-length PA-X prior to 1985, but since then SIVs with truncated PA-X have gradually increased and become dominant, implying that truncation of this protein may contribute to the adaptation of influenza virus in pigs. To verify this hypothesis, we constructed PA-X extended viruses in the background of a “triple-reassortment” H1N2 SIV with truncated PA-X, and evaluated their biological characteristics *in vitro* and *in vivo*. Compared with full-length PA-X, SIV with truncated PA-X had increased viral replication in porcine cells and swine respiratory tissues, along with enhanced pathogenicity, replication and transmissibility in pigs. Furthermore, we found that truncation of PA-X improved the inhibition of IFN-I mRNA expression. Hereby, our results imply that truncation of PA-X may contribute to the adaptation of SIV in pigs.

The natural reservoir of influenza A viruses is aquatic birds; however, some influenza strains have been spread and adapted stably in terrestrial birds or mammals^1^,^2^. The molecular mechanisms related to host adaptation of influenza virus are not yet fully elucidated. PA-X was recently identified as a fusion protein in influenza virus containing N-terminal 191 amino acids of PA protein and unique C-terminal region of 41 (truncation) or 61 (full-length) amino acids produced by +1 frameshift open reading frame (X-ORF) in gene segment 3^3^,^4^. Comprehensive evolution analysis has shown that the PA-X gene is conserved in influenza A viruses, which suggests that PA-X may have functional importance for influenza viruses^5^. In fact, several studies have demonstrated that PA-X is a virulence modulation factor of influenza A viruses^3^,^6^,^7^,^8^,^9^.

Genetic analysis indicated that the length of PA-X appears to be associated with viral lineages circulating in their particular hosts, implying species specificity of PA-X protein^5^. Avian, equine, and human seasonal H3N2 and H1N1 influenza viruses express a full-length PA-X protein 61-amino-acid product of X-ORF. By contrast, some influenza A viruses including, the 2009 pandemic H1N1, swine and canine viruses possess a stop codon (TAG) at the 42 position in the X-ORF sequence, which result in a truncated PA-X protein with 41 amino acids in the X domain^5^. In 1930, influenza viruses were first isolated from pigs and these viruses have become known as “classical” (CS) swine H1N1 influenza viruses (SIVs). CS SIV possessed a full-length PA-X until PA-X truncation occurred in 1985. Since then, CS SIVs with truncated PA-X gradually increased and replaced strains with full-length PA-X. Besides, the avian-origin gene segment 3 of “triple-reassortment” (TR) SIV expresses a truncated PA-X; in contrast, PA-X proteins from avian influenza viruses are full-length^5^. These facts suggest that SIV with truncated PA-X may potentially present a selective advantage in pigs.

To determine the effects of the truncation of PA-X for adaptation of SIV in pigs, we used reverse genetics to construct two TR swine influenza viruses, only differing in PA-X. These included truncated PA-X and full-length PA-X, and we compared their biological characteristics *in vitro* and *in vivo*.

## Results

### Prevalence of SIVs with truncated or full-length PA-X

**Figure 1:**
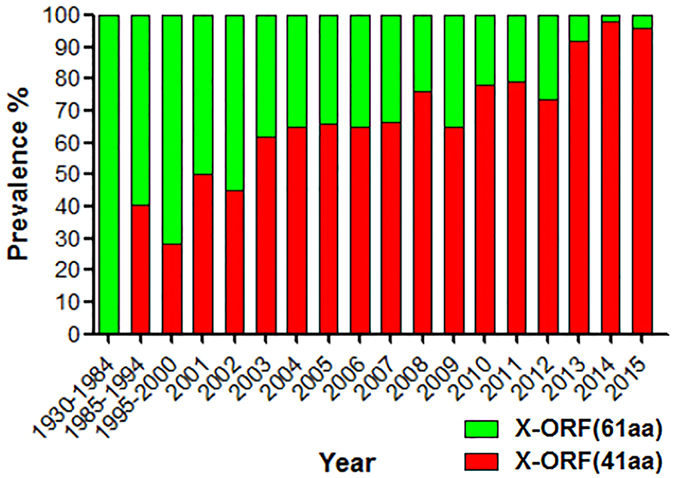
Prevalence of SIV with truncated or full-length PA-X. The proportion of swine influenza viruses with truncated or full-length PA-X was stratified by year. The numbers of PA sequences are as follows: 1930 to 1984, n = 144; 1985 to 1994, n = 78; 1995–2000, n = 79; 2001, n = 44; 2002, n = 40; 2003, n = 86; 2004, n = 94; 2005, n = 100; 2006, n = 86; 2007, n = 95; 2008, n = 100; 2009, n = 225; 2010, n = 258; 2011, n = 310; 2012, n = 342; 2013, n = 307; 2014, n = 138; 2015, n = 46.

To explore the relationship between truncation of PA-X and viral adaptation in swine, we analyzed the frequency of the PA-X truncations in SIV by year based on all SIV sequences available in the National Center for Biotechnology Information (NCBI) Influenza Viruses Resource, regardless of subtype and lineage. As shown in Fig. 1, the PA-X proteins in all SIV cases were full-length before 1985, following which the number of SIVs containing truncated PA-X gradually increased. By 2015, more than 90% SIVs possessed truncated forms of PA-X. These data suggested that the truncation of PA-X protein may play an important role in the adaptation of SIV in pigs.

### Generation of recombinant SIVs with truncated or full-length PA-X

**Figure 2:**
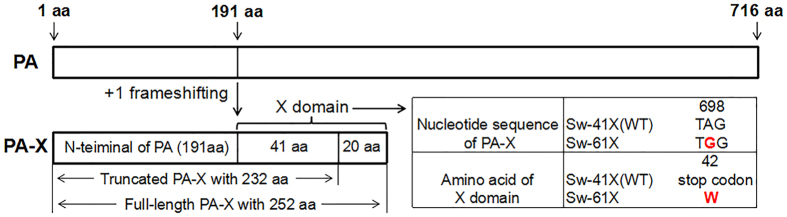
Schematic representation of PA and PA-X proteins. The PA-X encodes a common N-terminal domain (amino acid residues 1 to 191) of PA fused to a unique C-terminal region (41 or 61 amino acids) produced by a + 1 reading frame of PA mRNA via ribosomal frameshifting. The red letters represent mutation sites in PA-X to produce a full-length PA-X.

To investigate the effect of the length of PA-X on these biological properties, a reverse genetics system of wild type TR H1N2 SIV A/swine/Guangdong/1222/2006 [Sw-41X(WT)] possessing 41 amino acids in the X domain of PA-X was established as previously described^10^. Meanwhile, the X-ORF of Sw-41X(WT) virus was extended to the full-length 61 amino acids X domain by substituting the stop codon to tryptophan at the 42nd amino acid position in X-ORF without altering PA ORF (Fig. 2). This PA-X-extended H1N2 virus was named Sw-61X.

### *In vitro* properties of SIVs with truncated or full-length PA-X

**Figure 3:**
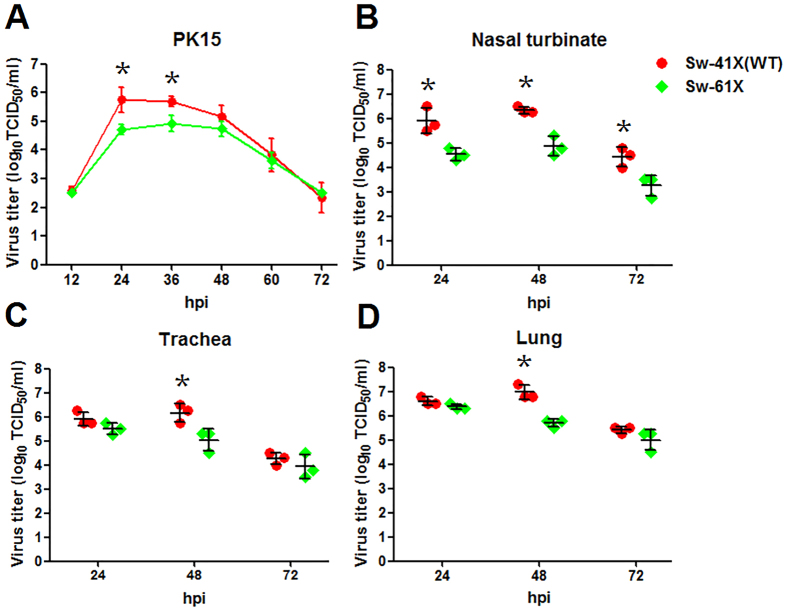
Viral growth kinetics of SIV in PK15 cells and porcine respiratory explants. (**A**) PK15 cells were infected with recombinant viruses at an MOI of 0.01 and supernatants were collected at indicated time. Nasal turbinate (**B**), trachea (**C**), and lung (**D**) explants were inoculated with 10^6^ TCID_50_ of each virus and supernatants were titrated. Data are presented as means ± SD of three independent experiments. *the value of Sw-41X(WT) was significantly different from Sw-61X (*P *< 0.05, ANOVA).

The influence of the length of PA-X on the replicative ability of H1N2 SIV *in vitro* was evaluated by inoculating porcine kidney (PK15) cells with each virus at a multiplicity of infection (MOI) of 0.01 in the presence of 1 μg/ml tosylsulfonyl phenylalanyl chloromethyl ketone-treated trypsin. As shown in Fig. 3A, the virus titers of Sw-41X(WT) were significantly higher than Sw-61X at 24 and 36 hours post-inoculation (hpi) (*P *< 0.05).

An *ex vivo* organ-culture model of the pig respiratory tract maintained at an air-liquid interface as a biologically relevant *in vitro* system was also used to study the replication of PA-X mutants as previously described^11^. Briefly, porcine nasal turbinate, trachea, and lung explants cultured in 12-well plates were inoculated with 10^6^ TCID_50_ of each virus, and virus titers in supernatants were subsequently tested at 24, 48, and 72 hpi. Sw-41X(WT) replicated more efficiently than Sw-61X in the explants of nasal turbinate at all three time points, and of trachea and lung at 48 hpi (*P *< 0.05) (Fig. 3B–D). Collected, these data indicated that truncation of PA-X promoted viral production in porcine cells or swine respiratory tissues infected *ex vivo*.

### Pathogenicity and transmissibility of SIVs with truncated or full-length PA-X in pigs

To determine whether truncation of PA-X influenced the pathogenicity and transmissibility of SIV in pigs, nine four-week-old landrace specific-pathogen-free piglets obtained from Beijing Center for SPF Swine Breeding & Management of each group were inoculated intranasally with 10^6^ TCID_50_ of each virus, and three infected pigs from each group were removed to a separate room with three naïve pigs at 24 hpi. The nasal turbinates, tracheas, and bronchoalveolar lavage fluids (BALFs) from three pigs in each inoculated group were collected for virus detection at 3 and 5 dpi. Clinical signs of the remaining three pigs were monitored daily throughout the duration of the study, and the nasal washes were collected at 3, 5, 7, and 9 days post-inoculation (dpi). Five naïve pigs were maintained as a control group and were mock-inoculated with 2 ml PBS, per nostril.

**Figure 4:**
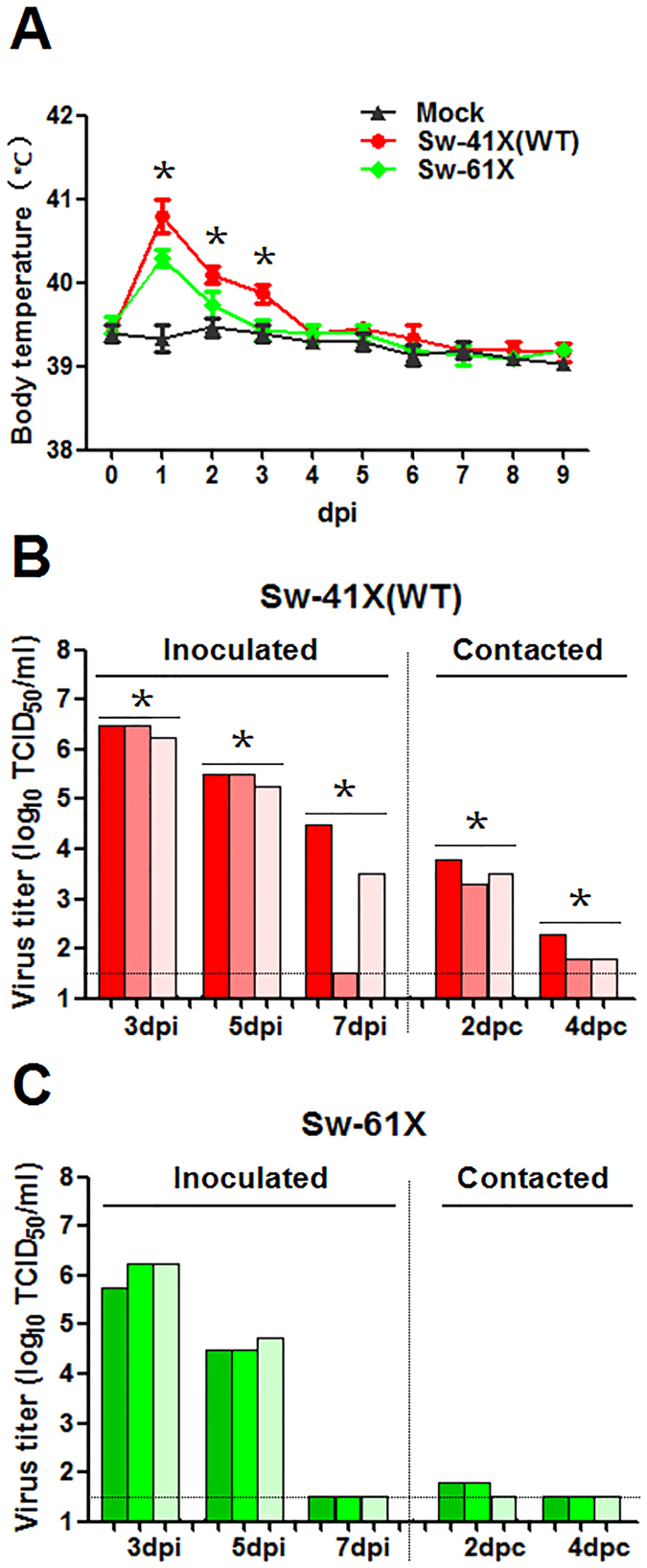
Body temperatures, virus shedding and transmission in pigs. Nine Pigs were intranasally inoculated with 10^6^ TCID_50_ of each virus. Five naïve pigs were maintained as a control group and were mock-inoculated with PBS. After 24 h, the inoculated animals were housed together with three contact pigs. Tissues from three pigs in each inoculated group were collected for virus detection at 3 and 5 dpi, respectively. (**A**) Rectal temperature of infected piglets was taken at fixed time-point daily from 0 to 9 dpi. (**B,C**) Viral titers in nasal washes of three infected or co-housed piglets from each group. Each bar represents the virus titer from an individual animal. The horizontal dashed line corresponds to the TCID_50_ assay detection limit. Data are presented as means ± SD of three pigs. *the value of Sw-41X(WT) was significantly different from Sw-61X (*P *< 0.05, ANOVA).

As shown in Fig. 4A, the mean rectal temperature in pigs inoculated with Sw-41X(WT) was significantly higher than that in Sw-61X infected pigs during 1 to 3 dpi (*P *< 0.05). Sw-41X(WT) caused lethargy and lack of appetite in two of three inoculated animals during 1 to 3 dpi, and nasal secretion and coughing were observed in one of them at 2 dpi. By contrast, infection with Sw-61X only produced slight lethargy and lack of appetite at 2 dpi in one infected animal. The virus titers of Sw-41X(WT) in nasal washes were significantly higher than Sw-61X at 3, 5 and 7 dpi (*P *< 0.05) (Fig. 4B,C). Furthermore, pigs infected with Sw-41X(WT) virus shed the virus for much longer than Sw-61X virus. At 7 dpi, Sw-41X(WT) was still detectable in two of three pigs with virus titers of 10^4.5^ TCID_50_/ml and 10^3.5^ TCID_50_/ml, respectively, but none in Sw-61X group had detectable virus by that time (Fig. 4BC). For the contacted animals, viral shedding was detectable in all the pigs co-housed with Sw-41X(WT) with titers of 3.5 ± 0.3 TCID_50_/ml and 1.9 ± 0.3 TCID_50_/ml at 2 and 4 days post-contact (dpc), respectively; while virus was only detectable in two of three pigs in Sw-61X group at 2 dpc with titers of 10^1.8^ TCID_50_/ml (Fig. 4BC). Seroconversion was only detected in animals with viral shedding. Overall, SIV with truncated PA-X had increased pathogenicity and transmissibility compared with that with full-length PA-X.

### Histopathology and immunohistochemistry of swine lungs infected by PA-X mutant viruses

**Figure 5:**
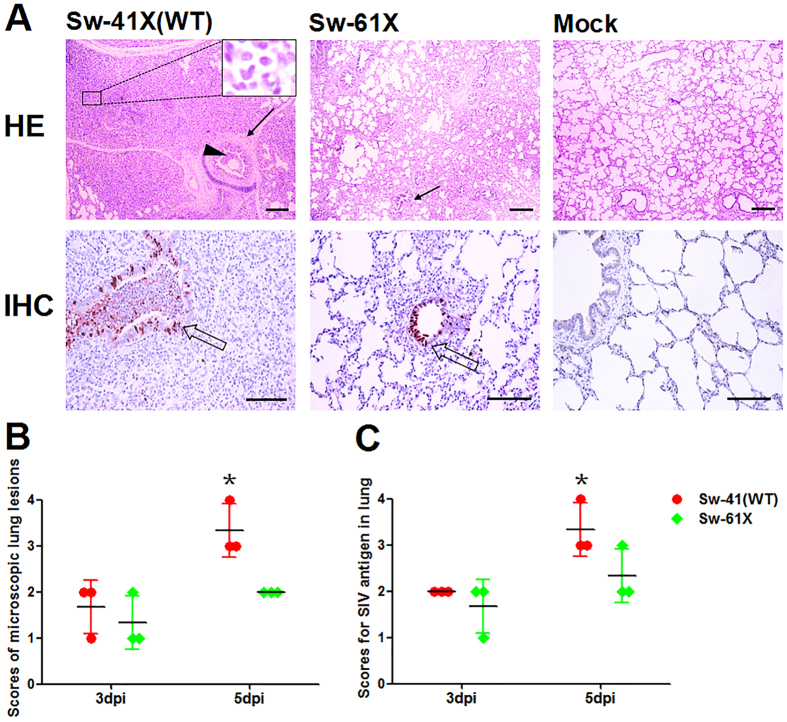
Pathological changes in the lungs of the inoculated pigs. (**A**) H&E and IHC examinations were performed on the lungs of pigs at 5 dpi. Solid arrows indicate edema of the bronchial and vessel walls around inflammatory cells, including lymphocytes and monocytes; triangle indicates desquamation of epithelial cells of the mucous membrane and diffuse infiltration of neutrophils and alveolar macrophages in the bronchial lumen. Open arrows show that SIV-positive signals were detected in the lungs of infected piglets. Scale bar, 200 μm. (**B,C**) Scores of microscopic lesions (**B**) and SIV-positive signals (**C**) in lungs were evaluated blinded by two independent pathologists. Data are presented as means ± SD of three independent pigs. *the value was significantly different from that of Sw-61X (*P *< 0.05, ANOVA).

To further examine viral pathology, three pigs from the infection group and one pig from the control group were anesthetized and their lungs were collected for evaluation of histopathology (H&E) and immunohistochemistry (IHC) at 5 dpi. Histopathological analysis showed that the Sw-41X(WT) virus caused moderate bronchopneumonia and consolidation as characterized by local edema, dropout of epithelial cells in bronchia, and infiltration of inflammatory cells; while only mild bronchopneumonia was observed for pigs infected by Sw-61X (Fig. 5A). Compared to Sw-61X, pigs exposed to Sw-41X(WT) showed increased SIV-positive signals in lungs (Fig. 5A). The average histopathological and immunohistochemical scores in the Sw-41X(WT) infected group were significantly higher than that of Sw-61X infected groups at 5 dpi (*P *< 0.05) (Fig. 5BC). No microscopic lesions and SIV-positive signals were observed in the lungs of control pigs (Fig. 5BC). Taken together, the above results further demonstrated that truncated PA-X increased the pathogenicity of SIV in pigs.

### Replication of PA-X mutant SIVs in pigs

**Figure 6:**
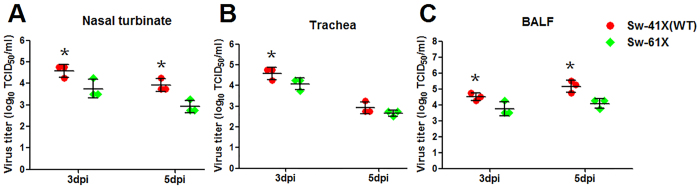
Viral replication in the tissues of the inoculated pigs. (**A–C**) Viral titers in nasal turbinates (**A**), tracheas (**B**) and BALFs (**C**) at 3 and 5 dpi. Data are presented as means ± SD of three pigs. *the value of Sw-41X(WT) was significantly different from Sw-61X (*P *< 0.05, ANOVA).

Pathogenicity experiments in pigs demonstrated that SIV with truncated PA-X presented higher pathogenicity than full-length PA-X SIV. To determine if the high pathogenicity was consistent with enhanced replication in tissues, virus titers in nasal turbinate, trachea, and BALFs were evaluated from three pigs in each group at 3 and 5 dpi. For the viral replication in the tissues of inoculated pigs, the replication titers of Sw-41X(WT) in nasal turbinates, tracheas and BALFs were significantly higher than Sw-61X at 3 dpi and/or 5 dpi (*P *< 0.05) (Fig. 6A–C). These results suggested that PA-X with 41 amino acids in X-ORF lead to increased viral replication *in vivo*.

### Inhibition of type I interferons expression by truncated or full-length PA-X

**Figure 7:**
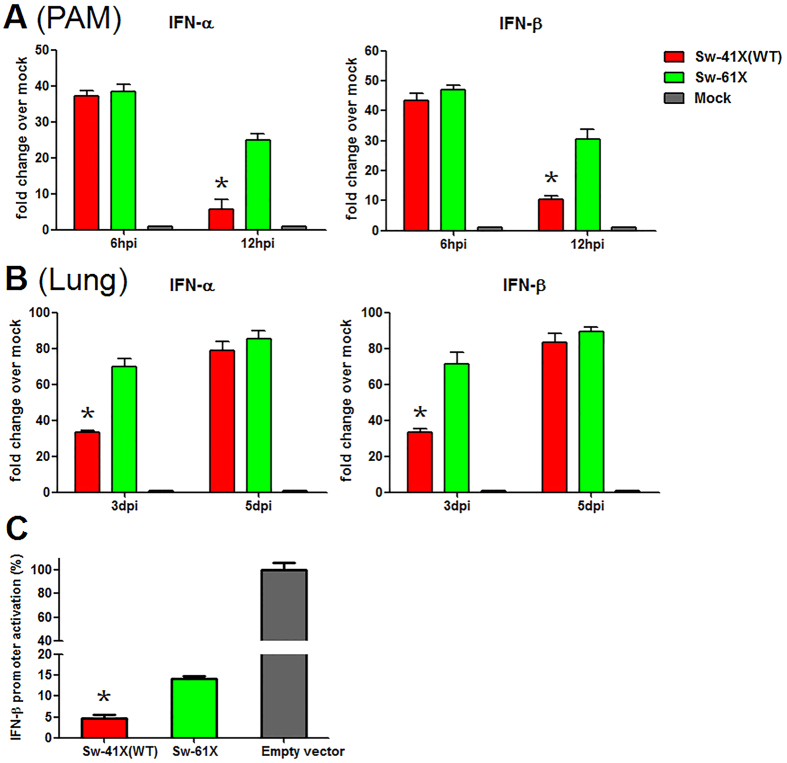
The effect of the length of PA-X in suppressing IFN-I expression. (**A**) Effect of PA-X on IFN-α and IFN-β mRNA expression in PAM cells, which were either uninfected or infected with SIV with truncated or full length PA-X at an MOI of 1.0. Total RNA was extracted from the infected cells at the indicated time points, the expression levels of IFN-α and IFN-β mRNA were quantified by real-time PCR. (**B**) IFN-α and IFN-β mRNA expression in lung homogenates at 3 dpi and 5 dpi. Total RNA was extracted from 100 μL of the homogenates, the expression levels of IFN-α mRNA and IFN-β mRNA were quantified by real-time PCR. Copy number in each sample was calculated using a standard curve of 10-fold serial dilutions of swine IFN-α or IFN-β gene product. Fold change indicates ratio of copy number of each sample over mock infection samples. (**C**) Luciferase reporter-mediated assay to quantify the PA-X protein inhibition effects on IFN-β promoter activation. Data are presented as means ± SD of three independent experiments. *the value of Sw-41X(WT) was significantly different from that of Sw-61X (*P *< 0.05, ANOVA).

We next explored the potential mechanism behind the enhanced replicatibility and transmissibility virulence of SIV caused by PA-X truncation. The induction of type I interferon (IFN-I) establishes an antiviral state which impedes viral replication and spread in host cells^12^. Recently, the PA-X protein of the influenza virus was demonstrated to be involved in the function of host shutoff which could inhibit IFN-I expression^9^. To assess whether the increased virulence of SIV with truncated PA-X was related to the increased inhibition of IFN-I expression, the expression levels of IFN-α and IFN-β mRNA in porcine alveolar macrophages (PAMs) and lungs of pigs infected with each virus were analyzed by quantitative real-time PCR as previously described. In PAMs, the expression levels of IFN-α and IFN-β mRNA from Sw-41X(WT)-infected cells were significantly lower than Sw-61X-infected cells at 12 hpi (*P *< 0.05) (Fig. 7A). In the lungs of infected pigs, Sw-41X(WT) virus induced a lower level of IFN-I expression than Sw-61X at 3 dpi (*P *< 0.05) (Fig. 7B).

To further confirm the direct effect of PA-X on IFN-I production, we expressed the truncated or full-length PA-X proteins ectopically in 293T cells and then infected them with Sendai virus to stimulate an innate immune response. Activation of IFN-β promoter was determined by a luciferase mediated reporter assay^13^. We found that IFN-β levels in cells expressing full-length PA-X was two-fold higher than cells expressing truncated PA-X (*P *< 0.05) (Fig. 7C). In general, these findings indicated that suppressing IFN-I response may contribute to the enhanced virulence of SIV with truncated PA-X.

## Discussion

Adaptation is thought to be the driving force in evolution, during which beneficial mutations are selected in nature because of increased replication and transmission in the new host. Pigs are proposed to be “mixing vessels” or opportune intermediate hosts for the generation of novel influenza viruses with pandemic potential^14^,^15^. Despite the pivotal role of pigs in the ecology of influenza A viruses, few virulence markers of influenza virus in pig are identified. Based on the fact that SIVs with truncated PA-X have been gradually increased and became dominan^5^, we investigate whether truncation of PA-X could adapt SIV for enhanced growth and transmission in pigs. We observed that truncation of PA-X promoted viral growth in PK15 cells and swine respiratory tissues infected *ex vivo*, increased the viral replication, pathogenicity and transmission in swine, and we further demonstrated that the altered ability of PA-X in suppressing IFN-I expression might responsible for these changes. These results suggested that SIV with truncated PA-X seemed to present a selective advantage in pigs, which is in accordance with the fact that the proportion of SIVs possessing truncated PA-X has continually increased and have become predominant in nature. In contrast, our previous study found that the 2009 H1N1, H5N1, and H9N2 influenza viruses with full length PA-X showed higher replication levels in A549 cells than those with truncated PA-X, and that virus with full length PA-X enhanced viral replication and pathogenicity in mice^16^. Coincidently, all of the avian and human influenza viruses possessed full-length PA-X in nature. Therefore, we speculate that the role of length of PA-X is associated with host specificity.

IFN-I consist of several structurally related IFN-α proteins and a single IFN-β protein, together with interferon-stimulated genes (ISGs), they establish an antiviral state in infected cells that function to inhibit viral replication and restrict viral spread^17^. To escape the antiviral response of the host, several viral proteins have been shown to suppress IFN-I expression, including vhs from herpes simplex virus (HSV) and nsp1 from severe acute respiratory syndrome-related coronavirus (SARS-CoV) or mouse hepatitis virus (MHV), that is necessary for efficient virus growth and virulence^18^,^19^,^20^,^21^,^22^,^23^,^24^,^25^,^26^. For the influenza virus, the newly identified PA-X protein, along with NS1, PB2, PB1, PA and PB1-F2 have been demonstrated to be involved in the IFN-I response modulation^27^. The most prominent IFN-I antagonist in influenza virus is the NS1 protein which can suppress the IFN-I response by interacting with RIG-I and TRIM25^28^,^29^,^30^. Graef *et al.* demonstrated that the PB2 subunit of the influenza virus RNA polymerase complex interacted with MAVS and inhibited MAVS-mediated IFN-β expression^31^. Additionally, the other polymerase proteins PB1 and PA were identified as part of the IFN-I inhibitory strategy evolved by influenza virus and suggested to interact with RIG-I, subsequently inhibiting RIG-I mediated IFN-I signaling^32^. Effects outlined of the PB1-F2 are strain specific, however interferon suppression seems to be a common property, as A/Hong Kong/156/1997 (H5N1) as well as PR8 (H1N1) strains both exhibit IFN-β antagonism^27^. More recently, Hayashi *et al.* reported that the presence of PA-X in A/California/04/2009 (H1N1) also resulted in a suppression of IFN-β mRNA production through the shutoff activity of PA-X, leading to more efficient viral replication and more severe lung pathology in infected cells and mice^9^. Here, we clearly showed that truncated PA-X was more effective in inhibiting IFN-I mRNA expression in PAMs and pigs through the shutoff activity of PA-X, which is consisted with the enhanced replication, pathogenicity and transmissibility of PA-X mutants in pigs. Therefore, our study provides evidence that the adaptation of SIV in pigs may be partly due to the shutoff activity of PA-X.

In summary, we found that truncation of PA-X played an important role in the adaptation of SIV in pigs. Compared with that of full length PA-X, it conferred increased viral pathogenicity, replication and transmissibility in pigs, promoted viral growth in PK15 cells and swine respiratory tissues infected *ex vivo*, and greater inhibition of host IFN-I expression, which was consistent with the increasing occurrence of SIV with truncated PA-X.

## Materials and Methods

### Ethics statement

All animal work was approved by the Beijing Association for Science and Technology (approval ID SYXK [Beijing] 2007–0023) and conducted in strict accordance with the Beijing Laboratory Animal Welfare and Ethics guidelines, as issued by the Beijing Administration Committee of Laboratory Animals, and in accordance with the China Agricultural University (CAU) Institutional Animal Care and Use Committee guidelines (ID: SKLAB-B-2010–003). The animal use protocol was approved by the Animal Welfare Committee of the CAU.

### Viruses and cells

A/swine/Guangdong/1222/2006 [Sw-41X(WT)] is a TR H1N2 swine influenza virus which has been previously described^10^. The Human embryonic kidney cell (293T), Madin Darby canine kidney cell (MDCK), porcine kidney cell (PK15), and porcine alveolar macrophages (PAMs) were kept in our laboratory. The 293T, MDCK, and PK15 cells were maintained in Dulbecco’s modified Eagle’s medium (DMEM, Gibco). The PAMs were maintained in RPMI 1640 medium (Gibco). The DMEM and RPMI 1640 media were supplemented with 10% fetal bovine serum (FBS, Gibco), 100 units/ml of penicillin, and 100 μg/ml of streptomycin; and all of the cells cultured at 37 °C in a 5% CO_2_ atmosphere.

### Generation of recombinant viruses by reverse genetics

All eight gene segments were amplified by reverse transcription (RT)-PCR from Sw-41X(WT) virus, and cloned into the dual-promoter plasmid pHW2000. The mutations were introduced into the PA gene using a Site-directed QuikChange Mutagenesis kit (Agilent) according to the manufacturer’s instructions. PCR primer sequences are available upon request. The TR H1N2 SIV with full-length PA-X (61aa), Sw-61X, was constructed by extending PA-X from 41aa to 61aa on the backbone of Sw-41X(WT) virus. Substitution from stop codon (UAG) to tryptophan (UGG) codon at position 42 in the X-ORF did not change the PA ORF (Fig. 2). Rescued viruses were detected using haemagglutination assays. Viral RNA was extracted and analysed by RT-PCR, and each viral segment was sequenced to confirm sequence identity.

### Viral titration and growth kinetics

The 50% tissue culture infectious dose (TCID_50_) was determined in MDCK cells with 10-fold serially diluted viruses inoculated at 37 °C for 72 h, as previous studies with Reed-Muench method^33^,^34^. Multistep replication kinetics was determined by inoculating PK15 cells with a MOI of 0.01. Supernatants were sampled at 6, 12, 24, 36, 48, 60, and 72 hours post-inoculation (hpi) and titrated by inoculating MDCK cells in 96-well plates. Three independent experiments were performed.

### Isolation, culture, and infection of porcine respiratory explants

Porcine nasal turbinate, trachea, and lung explants were prepared as described previously^11^,^35^,^36^. All respiratory explants were cultured at an air-liquid interface in 12-well plates at 37 °C in 5% CO_2_ atmosphere. At 24 h of culture, explants were washed with PBS, and 10^6^ TCID_50_ of each virus diluted in 500 μl explant medium were deposited in the upper compartment of Transwells for 1 h at 37 °C. Subsequently, explants were washed three times with PBS and the culture was replenished with 500 μl of explant media. At 24, 48, and 72 hpi, 100 μl supernatant was collected to assess virus yields.

### Infection and transmission of SIV in pigs

Four-week-old, landrace piglets that were free of influenza virus, porcine reproductive and respiratory syndrome virus (PRRSV), classic swine fever virus (CSFV), pseudorabies virus (PRV), porcine circovirus type 2 (PCV2) and *M. hyopneumoniae* infections were obtained from Beijing Center of SPF Swine Breeding & Management. Nine pigs from each group were inoculated intranasally with 10^6^ TCID_50_ of tested virus, delivered in a final volume of 2 ml per nostril, using a mucosal atomization device (MAD, Wolfe Tory Medical, Inc.) to mimic aerogenic infection. Five uninfected pigs were maintained as a control group and were mock-inoculated with 2 ml PBS per nostril, using the same method as the infected pigs. Three infected pigs from each group were removed to a separate room with three naïve pigs at 2 days post-inoculation (dpi). Clinical signs were observed by veterinary inspections at a fixed time daily throughout the duration of the study (14 days). Nasal washes were collected from all pigs at 3, 5, 7, and 9 dpi. At 3 and 5 dpi, three pigs from each infection group and one pig from control group were anesthetized and their nasal turbinates, tracheas, and lungs were collected for virus titration.

### Microscopical examination of lungs

The lung tissues collected at 5dpi were fixed in 10% phosphate-buffered formalin for histopathological examination which was performed as described previously^37^,^38^,^39^. Briefly, samples were processed for paraffin embedding and cut into 5-μm-thick sections. One section from each tissue sample was stained with hematoxylin and eosin (H&E) stain. Another section was processed for immunohistological staining with a rabbit polyclonal antibody (orb176401; Biorbyt, USA) against influenza A virus NP. Specific antigen-antibody reactions were visualized by using DAB Detection Kit (Polymer) (ORIGENE, Wuxi, China) and counterstained with hematoxylin.

The severity of microscopic lung lesions was scored by the distribution or by the extent of lesions within the sections examined as follows: 0, no visible changes; 1, mild focal or multifocal change; 2, moderate multifocal change; 3, moderate diffuse change; and 4, severe diffuse change^40^. The detection of swine influenza antigen in lung was executed through a ranked sore of 0–4, which was used to evaluate the number of positive cells per section taken from each block, as previously described^41^. The indication of the scores was as follows: 0, no SIV-antigen-positive cells; 1, 1–10 positive cells; 2, 11–30 positive cells; 3, 31–100 positive cells; and 4, = or > 100 positive cells. Two independent pathologists scored all lungs and slides from blinded experimental groups.

### Quantitative real-time PCR (qRT- PCR)

Total RNA in PAM cells or lung homogenates were extracted using TRIzol reagent at specified time points according to the manufacturer’s instructions (Invitrogen). Oligo-(dT) primed cDNA was generated from 1 μg of total RNA per sample using Superscript III First-Strand Synthesis SuperMix (Invitrogen). The qRT-PCR mixture for each sample consisted of 10 μl 2 × SYBR green PCR master mix (Applied Biosystems), 7 μl of nuclease-free water, 0.5 μl of each primer, and 2 μl of cDNA template. Amplification was performed using the 7500 real-time PCR system (Applied Biosystems) with the following program: 1 cycle at 95 °C for 10 min, followed by 40 cycles of 95 °C for 15 s and 60 °C for 1 min. Expression values for each gene, relative to RPL4, were calculated using the 2^−△△*CT*^ method. Each experiment contained three technical replicates for each sample, with two experimental replicates performed. Primers for qRT-PCR analysis of pig IFN-α/β are listed as following: IFN-α, Forward, 5′-GCCTCCTGCACCAGTTCTACA-3′, Reverse, 5′-TGCATGACACAGGCTTCCA-3′; IFN-β, Forward, 5′-TGCAACCACCACAATTCC-3′, Reverse, 5′-CTGAGAATGCCGAAGATCTG-3′.

### Interferon-β reporter assay

To measure the IFN-I shutoff function of PA-X, a luciferase based, Sendai virus-mediated IFN-β promoter activation assay was conducted as previously described^13^. Briefly, 293T cells in 24-well plates were transfected with 500 ng of empty vector or different PA-X proteins respectively. Additionally, 200 ng of an IFN-β-promoter-luciferase reporter plasmid (pIFNbLuc) and 20 ng of a plasmid constitutively expressing Renilla luciferase (pRL-TK from Promega) were transfected. At 18 hours post transfection, cells were infected with Sendai virus to induce the IFN-β promoter at a MOI of 5.0. At 18 hour post virus inoculation, cell lysate was prepared with Dual-Luciferase Reporter Assay System (Promega, USA) and luciferase activity was measured using GloMax 96 microplate luminometer (Promega, USA). The relative luciferase activity of the group with the empty vector was set as 100%, and the other groups were presented relative to that.

### Statistical analyses

All statistical analyses were performed using GraphPad Prism Software Version 5.00 (GraphPad Software Inc., San Diego, CA, USA). Statistically significant differences between experimental groups were determined using the analysis of variance (ANOVA) method. Differences were considered statistically significant at *P *< 0.05.

## Additional Information

**How to cite this article**: Xu, G. *et al.* Truncation of C-terminal 20 amino acids in PA-X contributes to adaptation of swine influenza virus in pigs. *Sci. Rep.* **6**, 21845; doi: 10.1038/srep21845 (2016).
